# MicroRNA as Candidate Biomarkers in Atypical Parkinsonian Syndromes: Systematic Literature Review

**DOI:** 10.3390/medicina58040483

**Published:** 2022-03-26

**Authors:** Anastasia Bougea

**Affiliations:** 1st Department of Neurology, Eginition Hospital, Medical School, National and Kapodistrian University of Athens, 72-74 Vassilisis Sofia’s Avenue, 11528 Athens, Greece; abougea@med.uoa.gr or annita139@yahoo.gr; Tel./Fax: +30-21-0728-9285

**Keywords:** multiple system atrophy (MSA), progressive supranuclear palsy (PSP), microRNAs (miRNAs/miRs), biomarkers

## Abstract

*Background and Objectives:* Multiple system atrophy (MSA) and progressive supranuclear palsy (PSP) are rare atypical parkinsonian syndromes, characterized by motor and cognitive symptoms. Their clinical diagnosis is challenging because there are no established biomarkers. Dysregulation of microRNAs (miRNAs/miRs) has been reported to serve an important role in neurodegenerative diseases. However, the miRNA profiles of MSA and PSP patients are rarely reported. The aim of this study was to critically review the role of miRNAs as diagnostic biomarkers to differentiate these atypical parkinsonian disorders and their role in disease pathogenesis. *Materials and Methods:* A systematic literature search of PubMed was conducted up to February 2022 according the Preferred Reporting Items for Systematic Reviews and Meta-Analyses (PRISMA) guidelines. *Results:* A total of 15 studies were analyzed. Three studies have shown that miR-9-3p, miR-19a, miR-19b, and miR-24 are potential biomarkers for MSA. In two studies, miR-132 was downregulated, whereas miR-147a and miR-518e were upregulated in the brain tissue of PSP patients. *Conclusions:* The potential of miRNA is still uncertain as a potential differential diagnostic marker to identify these disorders. Pre-analytical and analytical factors of included studies were important limitations to justify the introduction of miRNAs into clinical practice.

## 1. Introduction

Progressive supranuclear palsy (PSP) and multisystem atrophy (MSA) are rare atypical parkinsonian syndromes with heterogeneous clinical phenotypes, which makes their early diagnosis extremely difficult; 24% of these patients are misdiagnosed with idiopathic Parkinson’s disease (iPD) [1]. PSP is a debilitating tauopathy characterized by supranuclear palsy, postural instability with falls, and executive dysfunction early in the course of the disease. The diagnosis of PSP-P, the most common PSP variant, requires the presence of akinetic-rigid predominantly axial and levodopa resistant parkinsonism (A2) or parkinsonism with tremor and/or asymmetric and/or levodopa responsive (A3) [2]. MSA is a fatal synucleinopathy characterized by cerebellar, extrapyramidal, and pyramidal signs, along with dysautonomia, and relative preservation of cognitive functions. Conventionally, MSA is subclassified into a parkinsonian MSA-P with prominent symmetrical parkinsonism, and MSA-C with prominent cerebellar signs [3].

A biomarker is defined as a characteristic that can be objectively measured and evaluated as an indicator of normal biological processes, pathogenic processes, or pharmacological responses to a therapeutic intervention [4]. Given that (a) obtaining in vivo brain tissue samples is a highly invasive method and (b) many brain-related phenomena are reflected in the CSF, the latter could be an ideal source of biomarkers for detecting and monitoring various pathophysiological processes [5]. In Alzheimer’s disease (AD), for example, CSF biomarkers such as α-amyloid Aβ42, total Tau (Tau), and phosphorylated tau (pTau 181) reflect pathology in the AD brain and are now incorporated into the revised clinical diagnostic criteria for AD [6].

Despite the clear clinical differences in atypical parkinsonian syndromes, their differential diagnosis is difficult in clinical practice [7]. Early diagnosis is a key priority, as it helps to predict the course of the disease and the response to antiparkinsonian treatment. The main neuropathological feature of these diseases is the accumulation of proteins that could contribute to the degeneration of the dopaminergic system and other brain neurons [8]. The etiology has not been fully elucidated. Therefore, it is necessary to identify minimally invasive and disease-specific diagnostic biomarkers that will allow the early detection of pathological protein accumulation. To date, studies with protein biomarkers (α-synuclein, Aβ42, Tau, and pTau 181) in the CSF and blood of these patients have shown conflicting results [9,10,11].

MicroRNAs (miRs) are small, phylogenetically conserved RNA sequences (19–23 nucleotides) that inhibit protein expression by hybridizing to complementary sequences in the 3’ untranslated region (3’ UTR) of their target mRNAs [12]. To date, more than 2500 miRs have been identified in humans (miRbase). They have been associated with controlling important cellular processes, such as lipid metabolism, apoptosis, differentiation, and organ development. An altered miRNA expression has been linked to pathological features, such as inflammatory, degenerative, or autoimmune processes, and is involved in several diseases, including cancer, cardiovascular diseases, diabetes mellitus, and rheumatic and neurodegenerative diseases [13]. Importantly, miRNAs have recently been addressed as novel mediators of cell–cell communication, being cell-secreted, and are found in different biological fluids (blood, CSF, saliva, urine). Such characteristics also make miRNAs potential disease biomarkers in PD [14]. Several miRs have been linked to iPD as they regulate the expression of crucial proteins involved in pathophysiology, such as SNCA, leucine-rich repeat kinase 2 (LRRK2), GBA, and nuclear receptor related (NURR) -17. A recent meta-analysis of miRNAs quantified in brain (*n* = 125), blood (*n* = 31), or CSF (*n* = 4) resulted in 13 miRNAs, i.e., hsa-miR-132-3p (*p* = 6.37 × 10^−5^), hsa-miR-497-5p (*p* = 1.35 × 10^−4^), and hsa-miR-133b (*p* = 1.90 × 10^−4^), which are differentially expressed in the brain, and hsa-miR-221-3p (*p* = 4.49 × 10^−35^), hsa-miR-214-3p (*p* = 2.00 × 10^−34^), and hsa-miR-29c-3p (*p* = 3.00 × 10^−12^) in the blood of patients with PD [15]. Circulating brain-enriched miRNAs allow us to detect CNS signals from the peripheral blood circulation. For example, the discrimination accuracy of four miRNAs was moderate (AUC = 0.705), including miR-7, miR-124, miR129, miR-139, and miR-431, between PD and healthy controls [16]. Recently, we validated these findings in a larger sample of 109 patients with idiopathic PD and 92 healthy controls [17]. We showed the increased expression of miR22-3p, miR-139-5p, miR-154-5p, and miR-330-5p among patients with PD and healthy controls, and the highest discrimination accuracy was 0.730 by the pooled miRNAs including different miRNAs (miR-7-5p, miR-136-3p, and miR-409-3p). Of these, miR-22-3p, miR-124-3p, and miR-136-3p differed significantly between PD and healthy controls in previous studies [18,19,20,21], further confirming our findings.

The objective of this study was to conduct a first-time systematic review of the literature regarding the role of miRNAs as diagnostic biomarkers to differentiate these atypical parkinsonian disorders.

## 2. Materials and Methods

### 2.1. Study Design

A literature search was conducted in the MEDLINE database from inception until February 2022 by using the following search terms: “miRNA” OR “microRNA” AND “multiple system atrophy” OR “progressive supranuclear palsy”. I performed study selection according to the PRISMA (Preferred Reporting Items for Systematic Review and Meta-Analyses) guidelines for reporting systematic reviews [22]. To identify further potentially relevant studies missed by the database search strategy, the reference lists of retrieved articles were also manually screened.

### 2.2. Inclusion Criteria

A restriction was imposed during reference selection for human studies, animal, and cell culture studies published in English.

### 2.3. Exclusion Criteria

Studies were excluded if they (1) considered other parkinsonian syndromes than MSA and PSP; (2) were reviews, letters, editorials, abstracts or conference proceedings, or thesis papers.

## 3. Results

**Figure 1:**
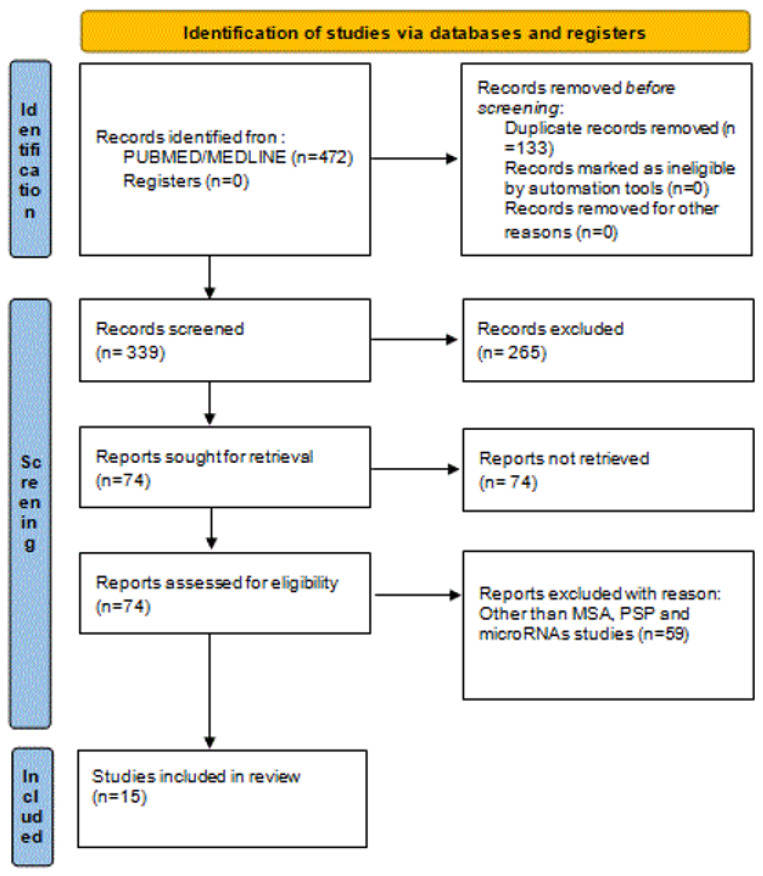
Identification of studies via using PRISMA flow diagram. Abbreviation: MSA: Multisystem Atrophy, PSP: Progressive supranuclear palsy.

Figure 1 represents the study selection and identification PRISMA flow diagram.

Out of 427 initially identified references through a database search, we obtained 74 references for further screening after initial title and abstract screening. All full-text articles of identified abstracts that met inclusion criteria (from inception until February 2022) were further scrutinized. In certain cases, the authors were asked to provide relevant data. Finally, 15 eligible studies were identified, which formed the basis of the present review. In this context, we reviewed the recent literature and we outline the altered miRNAs in MSA (12 studies) and PSP (3 studies) in Table 1 and Table 2.

### 3.1. MicroRNAs in MSA

Ubhi et al. [23] compared microRNA profiles between MSA patients and transgenic MSA muscle models with extensive deregulation of miRNAs. The miR-96 complex (miR-96, miR-182, and miR-183) was deregulated in both cases. These results are consistent with a recent study conducted by Vallelunga et al. [24]. Increased expression of miR-96 was observed, while SLC1A1 and SLC6A6 mRNA target genes and protein levels were decreased. Interestingly, miR-141 as a precursor to miR-141* was significantly altered, while let-7b and miR-141 were significantly deregulated in transgenic mice but not altered in MSA patients.

In blood, miRNA studies have also found variable results. Vallelunga et al. [25] analyzed 754 miRNAs in the serum of patients with MSA, PD, and healthy controls. They identified 12 new miRNAs, nine of which increased their expression (miR-29c, miR-24, miR-223*, miR-324-3p, miR-148b, miR-483-5p, miR-1291, miR-1274A, and miR-1274B), while their expression decreased in three (miR-339-5p, miR-652, and miR-744) in patients with MSA compared to healthy controls. In addition, comparison of miRNAs between PD and MSA-P showed increased expression of four microRNAs (miR-24, miR-34b, miR-148b, and miR339-5p) and decreased expression of miR-1274A. MiR-30c and miR-148b decreased in PD, while miR-148b increased in MSA (Table 1).

Similarly, using a microarray for serum samples, Kume et al. [26] found 67 dysregulated miRNAs; among these, miR-24 and miR-223 were found upregulated, as described by Vallelunga et al. [25]. There were 22 upregulated miRNAs and 17 downregulated miRNAs in MSA-P serum compared to MSA-C. Perez-Soriano [27] also replicated five miRNAs that had been initially reported by Kim et al. [28] to be DE in MSA striatum (miR-24-3p, miR-93-5p, miR-25-3p, miR-181a-5p, and let-7b-5p). Similarly, the functional analysis reported links with prion disease, fatty acid metabolism, and NOTCH signaling (implicated in oligodendrocyte demyelination).

Uwatoko et al. [29] presented opposing results after analyzing plasma rather than serum. In this case, after microarray analysis and RT-qPCR validation (of the top 11 DE miRNAs), miR-671-5p, miR-19b-3p, and miR-24-3p were found to be DE in MSA and PD. Conversely, miR-24-3p was found to be downregulated in MSA instead of upregulated. Similar differences were found among MSA-P and PD patients (miR-671 downregulated in both cases).

Μarques et al. [30] showed that the CSF serves as an excellent source for the study of miRNAs in patients with MSA, PD, and healthy controls. Two miRNAs (miR-24 and miR-205) differ significantly between PD and healthy controls and four miRNAs between MSA and healthy controls (miR-19a, miR19b, miR-24, and miR-34c). Combinations of miRNAs significantly differentiated PD or MSA patients from the control group. An interesting finding was that miR-24 and miR-148b were associated with cerebellar ataxia in MSA patients, suggesting that these miRNAs may be involved in the cerebellar neurodegeneration in MSA.

Schafferer et al. [31] studied miRNAs and their target expression in MSA mice models where α-Syn accumulation was previously present in oligodendrocytes in pathoanatomical preparations using RNA sequence and microarray. However, in this model, neither neuronal loss nor motor deficits resemble the clinical picture of MSA. The study showed, in the early stages of the disease, 59 different miRNAs in the substantia nigra and 33 in the striatum. Among them, miR433 with reduced expression in the striatum was involved in the regulation of HDAC6 expression, which is co-located with α-Syn in glial cytoplasmic inclusions of the brain with MSA. However, at this stage, the expression of HDAC6 has not changed. One explanation could be that, in the early stages of MSA, a change in miR-433 expression may precede HDAC6 expression, and this may play a key role in later stages of the disease.

Valera et al. [32] showed significantly increased expression of let-7b and miR-101, decreased expression of miR34c, and a tendency for increased expression of miR-30a, miR-96, and miR-183 in the striatum of a patient with MSA-P. Interestingly, the tendency for increased expression of miRNAs was significantly reduced in the striatum and no significant changes were observed in the cerebellum or frontal cortex, except for the decreased expression of microR-30a in the frontal cortex. Elevated miR-96 levels are consistent with the findings of Ubhi et al. [23]. MiR-183 plays a pivotal role in colorectal cancer progression, regulating UVRAG-mediated autophagy and apoptosis [33]. In addition, elevated miR-101 levels in the striatum of MSA-P patients, decreased expression of the predicted target genes of the RAB5A and mTOR (mammalian target of rapamycin) proteins, and increased α-Syn mRNA levels were found. This hypothesis was confirmed in an in vitro study where overexpression of miR-101 in CG-4 oligodendroglial cells led to α-Syn accumulation and autophagy deficits. Meanwhile, anti-miR-101 in both cellular and mice models resulted in reduced α-Syn accumulation in oligodendroglial cells and enhanced autophagy [34]. Furthermore, RAB5A is involved in the classification of intracellular vesicles during autophagy, and the inhibition of RAB5A and mTOR expression may affect protein clearance [35].

### 3.2. MicroRNAs in PSP

Only three studies are available on microRNAs and PSP. Smith et al. [36], using the TargetScan algorithm, explored potential binding sites for miRNA within the 3′ UTR of splicing agents that specifically regulate the splicing of exon 10 of tau protein. Overexpression of miR-9, miR-132, miR-124, miR-153, and miR-137 predicts the targeting of proteins bound to polypyrimidine region 1 and 2 (polypyrimidine tract-binding protein PTBP1 and PTBP2) in neuromuscular Neuro2A cells in which the expression 3R:4R-tau has been disturbed. Endogenous expression of these miRNAs increased relative 3R-tau levels, while miR-9, miR-137, and miR-132 decreased 4R-tau levels. Apparently, these miRNAs reduced the overall tau 4R:3R ratio. Counter information probes against these miRNAs reversed the 4R: 3R-tau ratio and their results. Interestingly, miR-137 and miR-9 increased total tau protein levels while miR-132 decreased them. This study demonstrates that miRNAs can regulate the abundance of tau isoforms. In addition, miR-132, miR-132*, and miR-212 were downregulated, while miR-9, miR-124, miR-137, and miR-153 remained unaffected in the brains of patients with PSP. MiR-132 targeted the PTBP2 protein, whose levels were elevated in patients with PSP. Overexpression of knockdown miR-132 or PTBP2 in neuronal cells similarly affected the exon 10 splicing of tau protein and the endogenous 4R:3R-tau ratio. In summary, changes in the miR132/PTBP2 pathway may contribute to the abnormal attachment of exon 10 of tau protein to the brains of these patients. Further characterization of miR-132 and PTBP2 in iPSC neuronal cells derived from PSP patients could shed light on the role of miRNAs in the pathogenesis of PSP (Table 2).

Tatura et al. [37] investigated the expression of 372 different miRs in the forebrain of 40 PSP patients and 40 controls using TaqMan and PCR techniques. The results showed significantly increased expression of miR-147a and miR-518e. In addition, this study confirmed that the target genes for miR-147a (ACLY, ALG12, and NF1) and miR-518e (JAZF1, CPEB1, and RAP1B) are repressed in the PSP. However, these findings do not concur with those of Smith et al., where miR-132 expression was significantly reduced in the temporal, parietal, and prefrontal sections of the brains of eight patients with PSP compared with eight healthy controls. In contrast, Tatura et al. showed only reduced expression of miR-132, although the difference with the control group was not significant, which could be attributed to the smaller sample size (*n* = 20). These studies suggest that miRNAs contribute to the pathogenesis of PSP by regulating the overexpression of neurotoxic proteins.

Nonaka et al. [38] used a microarray chip containing 2632 miRNAs to examine CSF miRNA expression levels in 11 patients with PSP and eight age-matched healthy controls. The miRNAs that were most significantly upregulated or downregulated in the early-onset group were miR-204-3p, miR-873-3p, and miR-6840-5p. The target genes of these miRNAs were associated with molecules related to the ubiquitin–proteasome system and autophagy pathway.

## 4. Discussion

There is limited evidence to date concerning the differential expression of miRNAs as diagnostic biomarkers for MSA and PSP. Three studies have shown that miR-9-3p, miR-19a, miR-19b, and miR-24 are potential biomarkers that can be used to distinguish patients with MSA from PD and healthy subjects [29,30,39]. However, there is little concordance in the miRNAs expressed in the CSF and peripheral blood of patients with MSA [25,30]. In two studies, miR-132 was downregulated, whereas miR-147a and miR-518e were upregulated in the brain tissue of PSP patients [36,37]. MiR-873-3p and miR-6840-5p were downregulated while miR-204-3p was upregulated in the CSF of the early-onset PSP group [38].

An important point in this review is the high degree of heterogeneity detected across the above studies. This could be explained by the small sample size and the lack of cross-validation between studies, making clinical applicability and data reliability very challenging. There were several methodological differences among the included studies, including differences in sample collection (post-mortem [40]) and storage, arrays or panels used, endogenous normalizers for validation, analytical software, statistical significance cut-offs, etc. Another factor is the intrinsic clinical heterogeneity of the disease or polyvalent nature of miRNAs [41]. RT-qPCR is considered the gold standard for accurate, sensitive microRNA detection among the other methods (NGS, microarrays). The miR-specific in-house qPCR method is a cost-effective alternative to more expensive commercial products for qPCR-based microRNA analysis; however, the majority of studies used only commercial products.

**Figure 2:**
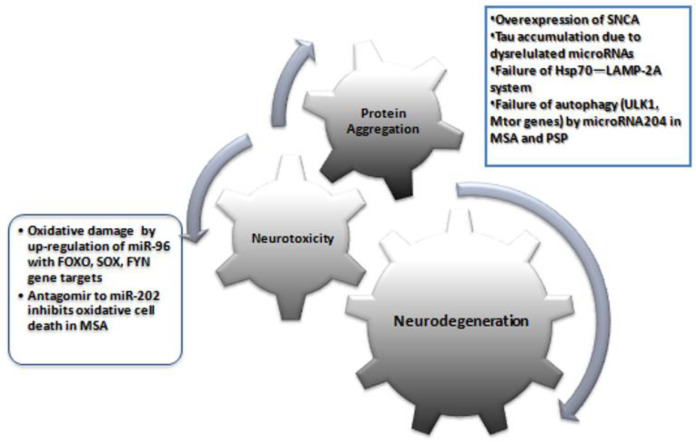
An overview of major pathogenetic mechanisms in atypical parkinsonian syndromes MSA and PSP and the proposed role of miRNAs.

There are a very limited number of studies available on PD from MSA patients. Vallelunga et al. [25] analyzed the expression of 754 circulating miRNAs in serum using TaqMan low-density array technology and found differentially expressed miRNAs in PD (four downregulated (miR-30c, miR-148b, miR-339-5p, miR-652), five upregulated (miR-24, miR-34b, miR-324-3p, miR223*, miR-1274A)) and MSA patients (three downregulated (miR-339-5p, miR-652, and miR-744), nine upregulated (miR-24, miR-29c, miR-148b, miR-223*, miR-324-3p, miR-483-5p, miR-1274A, miR-1274B, and miR-1291)) compared to healthy controls. Further, the evaluation of differentially expressed miRNAs between PD and MSA-P revealed five differentially expressed miRNAs. Importantly, miR-339-5p is downregulated in MSA cases, whereas miR-24, miR-223*, and miR-324-3p are upregulated in both MSA and PD. The present review has important clinical and research implications. Compared to protein biomarkers, miRNAs are advantageous because: (1) they are stable and resistant to RNase activity, as well as extreme pH and multiple numbers of freeze–thaw cycles; (2) they are not modified; (3) tissue-specific molecules; (4) preliminary studies showed little variation between sexes, age, or time of sampling, and (5) they can be easily and accurately measured with sensitive/specific laboratory protocols (e.g., RT-qPCR) [12,41]. Failure of protein homeostasis systems (Hsp70—LAMP-2A) and dysregulated autophagy-regulatory genes (ULK1, mTOR, Beclin-1, LC3, UVRAG, and Atg proteins) due to dysregulated miRNAs lead to protein aggregates in cells [42] (Figure 2).

Many of the genes targeted by miRNAs with significantly altered expression levels in this review were involved in different pathways. Dysregulation of miR-204 plays a role in the disruption of the autophagy pathway both in MSA and PSP [38,39]. Specifically for MSA, upregulation of miR-96 with gene targets, including FOXO, SOX, FYN, neuregulin, and SLC proteins (SLC1A1 and SLC6A6), could be involved in neurodegeneration, such as oxidative damage, zinc dyshomeostasis, and excitotoxicity [23]. Upregulation of miR-202 has been shown to downregulate the expression of OCT1, thus contributing to cerebellar degeneration in MSA [43]. Other miRNAs targeting genes have been reported to show epigenetic changes following EWAS of DNA from brain tissues of PSP patients. These results suggest that these miRNAs and their genes may be involved in the neurodegeneration of PSP and MSA. Future studies should focus on whether there is a specific microRNA molecular fingerprint for each parkinsonian syndrome and whether they point to specific molecular dysfunctions in neurons that can be used for target identification and tailored therapy.

Two promising miRNA-based therapeutic strategies are microRNA mimics and anti-microRNAs (antagomirs) [44]. MicroRNA mimics resemble miRNA precursors used to downregulate the expression of specific target proteins, whereas antagomirs are complementary RNA silencers particular to miRNAs and upregulate the expression of target proteins in vivo. This strategy may represent a novel therapeutic option for parkinsonian syndrome [45]. Lee et al. [43] showed that an antagomir to MiR-202 inhibited miR-202 enhanced oxidative stress-induced cell death. Apart from this study, none of the studies reviewed had used agomirs or antagomirs in MSA or PSP. More large-scale studies are needed to target protein or gene/s involved in the disease pathogenesis.

This review has its limitations. First, we could only investigate circulating microRNA I CSF, plasma, and serum due to the paucity of studies for other sample types. Second, there are a limited number of published studies on other parkinsonian or dementia syndromes for comparisons. Third, we only searched the PubMed/MEDLINE databases but we ensured that all available bibliography was included.

However, the present review also presents strong points: the literature was updated to comprise the latest evidence; limits of inclusion and exclusion criteria were defined for study selection.

## 5. Conclusions

Very limited evidence has been published on the microRNA differential expression in MSA and PSP, including different analytical approaches on different tissue types. Results, however, are inconsistent when aiming to identify specific differential targets. Despite recent advances, there remain several unanswered questions about the molecular pathogenesis of these diseases. The development of novel and robust technologies, such as patient-specific iPSC-derived dopaminergic neuronal miRNA profiling and characterization, will further the study of parkinsonian disorder at cellular and molecular levels. MicroRNAs could be used for prognosis and target therapy in parkinsonian syndromes through biotech companies that develop microRNA-based therapies. However, major challenges may remain as such for the time being, and miRNA-based diagnosis could be years away from clinical practice. Combining multiple microRNAs with novel statistical models such as machine learning may help to elucidate the pathological differences between MSA and PSP. Thus, these approaches will contribute to identifying biomarkers for the diagnosis of these atypical parkinsonian syndromes.

## Figures and Tables

**Table 1:** Overview of microRNAs in MSA patients and animal models.

Author	Sample	Biological Liquid/Tissue	Method	Upregulated miRNAs	Downregulated miRNAs	Sensitivity/ Specificity (%) AUC	Main Points
Vallelunga et al.	25PD, 25MSA, 25HC	serum	TaqMan Low Density Array	miR-24, miR-29c, miR-148b, miR223*, miR-324-3p, miR-483-5p, miR1274A, miR-1274B, and miR-1291	miR-339-5p, miR-652, and miR-744	NR	serum miRNA signatures discriminate PD from MSA and C
Marques et al.	28PD, 17MSA, 28C	CSF	RT-qPCR	miR-19a, miR-19b, miR-24, and miR34c	ns	MSA vs. C: 94/64 PD vs. MSA: 82/67	panels of miRNAs may be used as biomarkers of MSA
Ubhi et al.	Patients: 3MSA, 3AD, 3DLB, 3CBD, 3PSP, 4C	human + mice frontal cortex	RT-qPCR	miR-96, miR-182, and miR-183		NR	miR-96 target genes may be involved in MSA pathogenesis
Schafferer et al.	17MSA mice models + 14 C	SN and striatum of transgenic model	miRCURY LNA miRNA Array and RNA-seq	ns	miR-433	NR	early changes in the miRNA-mRNA regulatory network in the pathogenesis of MSA before the clinical onset of the disease
Wakabayashi et al.	13MSA, 13C	formalin-fixed paraffin-embedded MSA sample	miRCURY LNA Array	In pons (miR-1290, miR-21-5p, miR30b-5p, miR-4428, miR-23a-3p). In cerebellum (miR-4428, miR4732-5p, miR-1290, miR-3619-3p, miR4725-3p)	In pons (miR-128-3p, miR-371b-3p, miR3928-3p, miR-1915-3p, miR-129-2-3p, miR1203, miR-584-5p, miR-1910-5p, miR-675-5p, miR-149-5p, miR1233-3p, miR-3173-5p, miR-1539, miR-513a5p, miR-3663-5p, miR4723-3p, miR-4739, miR-4440, miR-1909-5p, miR-129-5p, miR330-5p, miR-572, miR4632-3p, miR-940,miR-1231, miR-124-3p, miR-34a-5p, miR-210-3p, miR-4687-5p, miR127-3p, miR-138-5p, miR-379-5p, and miR219a-5p) In cerebellum (miR-4739, miR-4726-3p, miR1228-3p, miR-346, miR-134-5p, miR-1233-3p, miR-484, miR-138-5p, miR-132-3p, miR3663-5p, miR-4440, miR-3184-5p, miR-557, miR-3907,miR-129-5p, miR-219a-2-3p, miR129-1-3p, and miR-129- 2-3p)	ΝR	postmortem samples from affected brain regions can be a valuable source for miRNA profiling in MSA
Valera et al.	17MSA, 7C	striatum	RT-qPCR	miR-183, miR-30a, miR-96, let-7b, and miR-101	miR-34c	NR	miR-101 dysregulation leads to MSA pathology mediated by autophagy deficit
Kume et al.	5MSA-P, 5MSA-P, 10HC	serum	Microarray	miR-16, miR451, miR-103a, miR223, miR-486-5p, miR-107, miR25, miR-3135b, miR15b, miR-185, miR939, miR-92a, miR4298, miR-92b, let-7c, miR-17, miR-4693-3p, miR-130a, let7d, let-7i, miR484, miR-4791, miR522, miR-26a, let7b, miR-3605-3p, miR-30d, miR4434, miR-4281, miR106a, miR-3667-3p, miR-99a, miR24, miR-221, miR31, miR-1285, miR218-2, let-7a, miR27a, miR-20a, miR518a-3p, miR19b, miR-10b, miR377, miR-4698, miR186, miR-126, miR1303, miR-500b, miR3622a-5p, mr3139	miR-4325, miR-380, miR3912, miR-4661-3p, miR4795-3p, miR-4458, miR3155, miR-590-3p, miR147b, miR-4439, miR378i, miR-3939, miR4495, miR-526b, miR548z, miR-3183	NR	These miRNAs may serve as biomarkers for MSA and contribute to the pathogenesis of MSA, through the accumulation of α-synuclein and the suppression of autophagy.
Lee et al.	4MSA, 4HC	cerebellum post-mortem	Microarray	miR-202 and miR199a-5p	miR-129-3p, miR-129-5p, miR-337-3p, miR-380, miR-433, miR-132, miR410, miR-206, and miR409-5p	NR	MiR-202 inhibited miR-202 enhanced oxidative stress-induced cell death
Uwatoko et al.	Study set: 11 MSA, 6 HC Validation set: 31 MSA-C, 30 MSA-P, 28 PD, 28 HC	plasma	3D-Gene^®^ Human miRNA oligo chip Ver. 17.0 (1720 miRNAs)	Study set: MSA vs. HC: miR-371b-5p, miR-4708-3p, miR-4736, and miR-663a) Validation set: miR-19b-3p στηνPD	Study set: MSA vs. HC: 75 miRNAs Validation set: miRNAs ns MSA vs. HC. MiR-671-5p 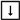 in PD vs. MSA-P; miR-24-3p 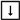 in MSA-C vs. PD	NR	hsa-miR-671-5p, hsa-miR-19b-3p, and hsa-miR-24-3p may reflect the pathophysiology or symptoms of PD and MSA.
Starhof et al.	Pilot cohort: 10 MSA, 10 PD, 10 HC, 10 PSP Validation cohort: 29 MSA, 37 PD, 32 PSP, 23 HC	CSF, EDTA, plasma	Pilot study: Exiqon miRCURY PCR Panel I version IV (372 miRNAs) Validation study: Fluidigm Biomark RTqPCR system (46 miRNAs)	All patient group CSF: let-7b-5p, miR-106b5p, miR-184, miR-218-5p, miR-331-5p, miR-34c-3p, miR-7-5p, and miR-99a-5p. Plasma: miR-218-5p, miR574-3p, miR-191-5p, miR30c-5p, and miR-873-3p		AUC (CSF) MSA vs. C:0.87 MSA vs. PD:0.73 PD vs. PSP:0.85 AUC (plasma) MSA vs. C: 0.85 MSA vs. PD: 0.78 PD vs. PSP:0.71	3CSF-microRNAs discriminate well PD, MSA vs. C 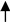
Vallelunga et al.	51 PD, 52 MSA, 56 HC	serum	qRT-PCR	MSA vs. HC: miR-96-5p PD vs. MSA: miR-339-5p	MSA vs. HC: miR-339-5p	NR	serum miR-96-5p in MSA vs. C
Perez-Soriano et al.	Discovery set: 7 MSA-C, 13 MSA-P, 19 PD, 40 HC Validation set: 8 MSA-C, 12 MSA-P, 18 PD, 40 HC	serum	GeneChip miRNA 4.0 array (2025 miRNA). FC > 11.51 *p* < 0.05	hsa-mir-16-5p, hsa-mir-191-5p, hsa-mir-24-3p, hsa-mir-7641, hsa-let-7b-5p, hsa-mir-425-5p, hsa-mir-23a-3p, hsa-mir-93-5p, hsa-mir-122-5p, hsa-mir-103a-3p, hsa-mir-4530, hsa-mir-17-5p, hsa-mir-140-3p, hsa-mir-106a-5p, hsa-mir-107, hsa-mir-25-3p, hsa-mir-7704, hsa-mir-181a-5p, hsa-mir-4487	hsa-mir-6797-3p, hsa-mir-940, hsa-mir-6796-3p, hsa-mir-3648, hsa-mir-1225-5p, hsa-mir-3197	NR	miR-7641 and miR-191 differentiate MSA from PD

AUC: area under the curve, CSF: cerebrospinal liquid, EDTA: ethylenediaminetetraacetic acid, HC: healthy control, MSA: multiple system atrophy, ns: non-significant, NR: non reported, PD: Parkinson’s disease, P: MSA-Parkinsonian variant, C: MSA-Cerebellar variant, SN: substantia nigra, PSP: progressive supranuclear palsy.

**Table 2:** Overview of microRNAs in PSP patients and animal models.

Authors	Sample	Biological Fluid/Tissue	Method	Upregulated miRNAs	Downregulated miRNAs
Smith et al.	8PSP, 8C	Human temporal, parietal, and prefrontal lobes	RT–PCR	ns	miR-132 (and their cluster miR-132, miR132*, and miR-212)
Tatura et al.	40PSP, 40C	Human inferior frontal gyri	qRTPCR	miR-147 (miR-147a) and miR-518e	miR-132
Nonaka et al.	11PSP, 8HC	CSF	3D-Gene 3000 miRNA microarray	hsa-miR-204-3p, hsa-miR-4476, hsa-miR-6132, hsa-miR-4638-5p, hsa-miR-7110-5p, hsa-miR-3679-5p, hsa-miR-1236-5p, hsa-miR-6867-3p, hsa-miR-6761-3p, hsa-miR-423-5p, hsa-miR-7111-3p, hsa-miR-3156-3p, hsa-miR-12114, hsa-miR-6889-5p, hsa-miR-6740-3p, hsa-miR-885-5p, hsa-miR-6894-3p, hsa-miR-487b-5p, hsa-miR-6820-5p, hsa-miR-873-3p, hsa-miR-7109-3p, hsa-miR-5193, hsa-miR 4648,hsa-miR-10398-5p, hsa-miR-1825,hsa-miR-6870-5p, hsa-miR-6825-5p, hsa-miR-4700-3p, hsa-miR-3622a-3p, hsa-miR-5001-5p, hsa-miR-6510-5p, hsa-miR-4505, hsa-miR-4665-5p, hsa-miR-8485, hsa-miR-7110-3p, hsa-miR-6862-3p, hsa-miR-6886-3p, hsa-miR-328-5p	hsa-miR-6840-5p

CSF: cerebrospinal liquid, HC: healthy control, MSA: multiple system atrophy, ns: non-significant, PSP: progressive supranuclear palsy.

## Data Availability

Not applicable.
